# The burden of suicide in Brazil: findings from the Global Burden of Disease Study 2019

**DOI:** 10.1590/0037-8682-0299-2021

**Published:** 2022-01-28

**Authors:** Cecília Silva Costa Bonadiman, Mohsen Naghavi, Ana Paula Souto Melo

**Affiliations:** 1 Universidade Federal de Minas Gerais, Faculdade de Medicina, Programa de Pós-Graduação em Saúde Pública, Belo Horizonte, MG, Brasil.; 2 Institute for Health Metrics and Evaluation, Seattle, WA, USA.; 3 Universidade Federal de São João Del Rei, Faculdade de Medicina, Divinópolis, MG, Brasil.

**Keywords:** Suicide, External causes, Mortality, Years of life lost due to premature death, Descriptive epidemiology

## Abstract

**INTRODUCTION:**

Suicide deaths varies according to location, sex, and age. This study analyzed the Global Burden of Disease Study 2019 (GBD 2019) concerning suicide in Brazil.

**METHODS:**

This study described the mortality and years of life lost (YLL) due to premature death caused by suicide in Brazil in 1990 and 2019. The numbers, crude and age-standardized mortality rate (ASMR), and YLL were compared among Brazilian states, age groups, and sexes.

**RESULTS:**

There were 13,502 suicides in Brazil in 2019, 46.00% more than in 1990. The crude mortality rate increased 0.32%, while the ASMR declined -21.68% during the period. Crude and age-standardized YLL rates declined by -7.24% and -18.38%, respectively. In 2019, the biggest ASMRs were found in the South, whereas from 1990 to 2019, the ASMR declined in the South, Southeast, and Midwest, and increased in the Northeast. The number of suicides was higher among individuals aged 15-49 years, and suicide rates were higher among those aged over 70 years. From 1990 to 2019, an increase in the rate was found only of 10-14 years of age. Suicide was highest in men, except in the 10-14-year age group, ranking third in mortality among men of 15-34 years of age and fourth among women of 15-24 years of age.

**CONCLUSIONS:**

The ASMR and YLL for suicide declined since 1990, but suicide remains an important factor of mortality in the country. The South Region, men, elderly, and youth should be priorities in the implementation of suicide prevention strategies in Brazil.

## INTRODUCTION

Suicide is defined as death caused by self-harm with intent to die^1^. Suicide ranks second as a cause of death among individuals of 15 to 29 years of age worldwide, second only to road traffic injuries^2^. The fact that suicide is a preventable cause of death, with significant emotional and economic impacts^3^, led the United Nations (UN) to include the reduction of suicide mortality rates by one third from 2015 to 2030 in its Sustainable Development Goals (SDG)^4^.

According to the Global Burden of Disease (GBD) study, the number of deaths from suicide was 817,000 globally (95% uncertainty interval [95% UI]: 762,000 to 884,000) in 2016, a 6.7% increase (95% UI: 0.4% to 15.6%) compared to 1990. However, the age-standardized suicide rate decreased by 32.7% (95% UI: 27.2% to 36.6%) in the same period, with expressive differences among countries^5^. Despite this, current trends suggest only 3% of the 188 countries will achieve the SDG target set for suicide mortality by 2030^6^.

The overall age-standardized suicide rate ranged from 27.5 (95% UI: 20.1 to 37.2) in Eastern Europe to 4.8 (95% UI: 4.2 to 5.6) in North Africa and the Middle East in 2016. The age-standardized suicide rate in Brazil was 6.4 (95% UI: 5.3 to 7.9), considerably less than rates reported in the United States of America (US) (12.9; 95% UI: 10.9 to 14.4), Chile (10.9; 95% UI: 7.9 to 15.4), and Argentina (11.2; 95% UI: 9.2 to 12.9)^5^. 

Brazil is a vast country with significant cultural, social, and economic diversity among its states. Therefore, risk factors for suicide are not homogeneously distributed throughout the country. Investigation of suicide rates in each state is necessary to identify more vulnerable subpopulations and support effective policies and suicide prevention programs. 

Nonetheless, suicide death records are inaccurate for several reasons, from social stigmatization to difficulties in establishing intentionality^7^, and suicide death is often coded as death of undetermined causes, by drowning or accidental poisoning^3^ ^,^ ^7^. Hence, the GBD study has proposed a method to reclassify ill-defined causes of death as other causes, such as suicide. The application of standardized methods to estimate suicide mortality enables a comparative analysis of the data by location, sex, and age group^8^ ^,^ ^9^. In this article, 2019 GBD estimates were used to describe the estimates of suicide mortality in Brazil and respective states, according to age and sex, in 1990 and 2019.

## METHODS

Data extracted from the secondary database of the 2019 GBD were used to analyze Brazilian suicide mortality rates. The 2019 GBD study is coordinated by the Institute for Health Metrics and Evaluation (IHME), of the University of Washington (USA), which has provided estimates for 369 diseases and injuries, together with 87 risk factors, in 204 countries^8^. In Brazil, metrics were estimated per each of the 27 states. 

Brazilian suicide mortality data reported in the 2019 GBD study were obtained from Mortality Information System (SIM, in Portuguese) of the Brazilian Ministry of Health. Suicide mortality was defined according to the International Statistical Classification of Diseases and Related Health Problems - 10^th^ revision (ICD-10)^10^ as death caused by intentional self-poisoning or self-harm (ICD-10 codes X60- X64.9, X66-X84.9, Y87.0; ICD-9 codes E950-E959). 

Methods of suicide were not specified in this study. Given the low suicide rates and the difficulty to determine intentionality of the act in children, deaths by suicide were investigated in individuals aged over 10 years of age^5^. 

For mortality estimation purposes, the GBD study data are corrected for the under-reporting of deaths, and deaths due to ill-defined cause or assigned to garbage codes are redistributed to defined causes (cause grouping methods have been detailed elsewhere)^9^. Undercounting or wrong assignment of death to suicide is common. To mitigate the effects of the wrong assignment of death from suicide, a fraction of deaths coded as undetermined intent injury (codes Y10-Y34 in ICD-10; E980-E988 in ICD-9), exposure to unspecified factors (codes X59 in ICD-10; E887 in ICD-9), or as poorly defined or unknown causes of mortality (R99) were redistributed to suicide^5^. 

GBD estimates are generated using statistical methods and modelling. Most cause of death (including suicide) estimates were modeled using the Cause of Death Ensemble Model (CODEm). This method is used to generate indicators by age, sex, country, year, and cause. CODEm is an analytical instrument that tests several possible statistical models of causes of death, which are then combined into an ensemble of models with optimal predictive performance. Detailed description of methods used to estimate suicide mortality has been detailed elsewhere^9^.

Aside from mortality, the GBD study provides estimates of years of life lost (YLL) due to premature death. This metric accounts for age at death and expresses the effect of premature death in the population, providing a broader overview of the burden of suicide. YLL is calculated as the number of deaths due to a cause-specific in each age group multiplied by the difference between the age at which death occurs and the standard life expectancy at that age. Standard life expectancy was based on the lowest observed risk of death in each 5-year age group, in populations greater than 5 million individuals. Further details about these calculations are available^9^.

In this study, Brazil and its states’ suicide mortality and YLL data collected in 1990 and 2019 were presented by sex and age group. The comparison of rates showing the temporal change was evaluated based on the difference between the rates in the time periods, in terms of percentage. GBD presents UI of the temporal change, which is considered significant when this UI does not include zero. 

Crude and age-standardized mortality rates per 100,000 inhabitants were provided. Crude mortality rates express non-adjusted data and are relevant for managers, whereas age-standardized mortality rates allow for data comparison over time and across different locations, following adjustments for age stratification differences in the population.

Age-standardized mortality rates were calculated by direct standardization using the global GBD study population. Intervals of uncertainty (95% UI) were provided for all estimates^9^.

The GBD Brazil study was approved by the Research Ethics Committee of the Federal University of Minas Gerais (UFMG, in Portuguese) (logged under CAAE Project number - 62803316.7.0000.5149).

## RESULTS

In 2019, there were 13,503 (95% UI: 12,815 to 14,735) suicide deaths in Brazil. Of these, 2,728 (95% UI: 2,551 to 3,025) involved women and 10,774 (95% UI: 10,152 to 11,953) involved men. For both sexes combined, deaths by suicide accounted for 0.96% (95% UI: 0.92% to 1.04%) of all deaths in the country. From 1990 to 2019, the overall number of deaths and the suicide crude mortality rate increased by 46.00% (95% UI: 37.20 to 59.87) and 0.32% (95% UI: -5.75 to 9.82), respectively. However, the age-standardized mortality rate for suicide decreased by -21.68% (95% UI: -26.30 to -14.28), from 7.25 (95% UI: 7.05 to 7.50) to 5.68 (95% UI: 5.40 to 6.19) deaths per 100,000 inhabitants in 1990 and 2019, respectively (Table 1).

**TABLE 1:** Total number of deaths, crude mortality rate (MR), and age-standardized mortality rate (ASMR) due to suicide in 2019, total percent change from 1990 to 2019, and ratio of female to male ASMR, for Brazil and its states.

Location	Number of deaths	% change number	MR	% change	ASMR	% change	Female to
	(95% UI)	of deaths 1990-2019	(95% UI)	MR 1990-2019	(95% UI)	ASMR 1990-2019	male ASMR ratio
Global	759 028	2.70	9.81	-28.99	9.39	-38.91	2.31
	(685 390 to 831 856)	(-5.93 to 13.12)	(8.86 to 10.75)	(-34.95 to -21.8)	(8.48 to 10.29)	(-43.93 to -32.71)	
Brazil	13 503	46.00	6.23	0.32	5.68	-21.68	4.24
	(12 815 to 14 735)	(37.20 to 59.87)	(5.91 to 6.80)	(-5.75 to 9.82)	(5.4 to 6.19)	(-26.30 to -14.28)	
South region							
Rio Grande do Sul	1 231	20.10	10.90	-1.65	9.10	-25.3	4.18
	(1 070 to 1 419)	(4.42 to 38.97)	(9.47 to 12.56)	(-14.50 to 13.80)	(7.93 to 10.51)	(-35.07 to -14.02)	
Santa Catarina	649	62.17	9.08	3.15	7.87	-26.67	3.94
	(571 to 754)	(41.35 to 92.27)	(7.99 to 10.54)	(-10.09 to 22.30)	(6.93 to 9.12)	(-36.14 to -13.39)	
Paraná	815	22.75	7.16	-7.63	6.34	-30.33	4.27
	(707 to 931)	(6.18 to 40.94)	(6.21 to 8.18)	(-20.10 to 6.06)	(5.51 to 7.25)	(-39.66 to -19.76)	
Southeast region							
Minas Gerais	1 633	46.04	7.53	7.55	6.63	-16.72	4.04
	(1 439 to 1 864)	(26.42 to 68.48)	(6.64 to 8.59)	(-6.90 to 24.07)	(5.86 to 7.55)	(-27.96 to -4.10)	
São Paulo	2 571	31.64	5.65	-7.45	4.98	-25.3	4.09
	(2 260 to 2 939)	(13.89 to 55.68)	(4.97 to 6.46)	(-19.93 to 9.45)	(4.38 to 5.71)	(-35.18 to -11.98)	
Espírito Santo	204	86.62	5.16	23.54	4.62	-6.28	3.67
	(177 to 233)	(59.68 to 115.31)	(4.47 to 5.86)	(5.71 to 42.53)	(4.01 to 5.25)	(-18.85 to 7.44)	
Rio de Janeiro	724	-39.51	4.10	-55.27	3.55	-61.51	3.77
	(645 to 817)	(-46.99 to -29.60)	(3.65 to 4.62)	(-60.8 to -47.94)	(3.16 to 3.99)	(-66.16 to -55.22)	
Midwest region							
Mato Grosso do Sul	217	50.25	7.65	-5.20	7.10	-27.52	3.89
	(188 to 251)	(29.57 to 76.87)	(6.63 to 8.87)	(-18.25 to 11.59)	(6.15 to 8.21)	(-37.66 to -15.39)	
Goiás	526	25.36	7.65	-24.49	6.91	-42.11	3.93
	(437 to 627)	(0.88 to 57.48)	(6.36 to 9.12)	(-39.24 to -5.14)	(5.77 to 8.22)	(-53.57 to -27.70)	
Mato Grosso	196	132.39	5.45	28.19	5.05	-10.16	3.69
	(171 to 224)	(89.66 to 196.08)	(4.77 to 6.24)	(4.62 to 63.32)	(4.44 to 5.78)	(-25.32 to 11.86)	
Distrito Federal	125	54.65	4.16	-17.54	3.86	-37.48	3.78
	(109 to 147)	(31.25 to 86.09)	(3.62 to 4.86)	(-30.02 to -0.78)	(3.37 to 4.49)	(-46.78 to -24.93)	
Northeast region							
Ceará	802	181.75	8.00	82.12	7.65	42.74	4.77
	(665 to 970)	(117.64 to 268.26)	(6.63 to 9.67)	(40.68 to 138.04)	(6.35 to 9.23)	(9.42 to 88.06)	
Piaui	277	152.91	7.50	81.24	7.30	31.58	4.17
	(242 to 314)	(111.67 to 200.94)	(6.58 to 8.51)	(51.68 to 115.65)	(6.39 to 8.28)	(10.55 to 56.54)	
Rio Grande do Norte	247	91.97	6.63	26.33	6.24	-2.68	4.73
	(204 to 295)	(52.88 to 139.09)	(5.46 to 7.89)	(0.61 to 57.34)	(5.13 to 7.42)	(-22.40 to 21.43)	
Sergipe	157	88.73	6.54	17.14	6.16	-14.03	4.12
	(133 to 186)	(54.48 to 128.96)	(5.55 to 7.76)	(-4.12 to 42.100	(5.24 to 7.30)	(-29.94 to 4.37)	
Pernambuco	564	52.78	5.58	10.14	5.31	-14.83	4.03
	(490 to 641)	(29.47 to 76.98)	(4.85 to 6.33)	(-6.66 to 27.59)	(4.61 to 6.01)	(-27.95 to -1.32)	
Paraíba	237	129.00	5.41	70.71	5.15	32.9	4.08
	(203 to 274)	(84.34 to 174.26)	(4.64 to 6.25)	(37.42 to 104.44)	(4.42 to 5.94)	(7.92 to 59.72)	
Bahia	869	204.02	5.45	130.96	5.13	65.89	5.39
	(712 to 1 045)	(139.57 to 282.53)	(4.47 to 6.55)	(81.0 to 190.60)	(4.22 to 6.16)	(30.98 to 108.90)	
Maranhão	380	72.62	4.55	4.33	4.93	-19.28	4.46
	(310 to 460)	(25.96 to 146.47)	(3.71 to 5.51)	(-23.87 to 48.96)	(4.02 to 5.95)	(-41.11 to 17.71)	
Alagoas	140	104.27	3.83	42.69	3.81	9.24	3.54
	(121 to 162)	(67.44 to 146.21)	(3.31 to 4.44)	(16.96 to 71.99)	(3.29 to 4.4)	(-9.56 to 31.95)	
North region							
Roraima	45	210.40	7.57	8.09	7.91	-22.55	3.69
	(40 75 to 50 75)	(159.30 to 290.41)	(6.8 to 8.47)	(-9.71 to 35.95)	(7.12 to 8.82)	(-33.49 to -7.30)	
Tocantins	104	207.15	6.37	70.99	6.26	18.54	4.25
	(87 to 123)	(143.50 to 286.74)	(5.34 to 7.53)	(35.56 to 115.30)	(5.25 to 7.39)	(4.63 to 47.00)	
Rondônia	115	64.66	6.50	2.59	6.10	-31.49	4.42
	(98 27 to 134 38)	(30.22 to 118.79)	(5.53 to 7.57)	(-18.86 to 36.33)	(5.20 to 7.07)	(-44.69 to -13.49)	
Acre	55	186.60	5.94	28.30	6.09	-4.89	5.19
	(48 to 62)	(139.08 to 236.56)	(5.22 to 6.77)	(7.03 to 50.66)	(5.35 to 6.92)	(-19.15 to 10.04)	
Amapá	49	359.82	5.82	47.41	5.82	6.26	4.48
	(44 to 54)	(292.58 to 457.48)	(5.25 to 6.43)	(25.85 to 78.72)	(5.26 to 6.43)	(-7.66 to 23.92)	
Amazonas	217	167.61	5.15	31.55	5.15	-4.25	4.85
	(190 to 248)	(123.98 to 222.97)	(4.52 to 5.9)	(10.10 to 58.76)	(4.54 to 5.92)	(-19.04 to 13.83)	
Pará	341	109.24	3.70	10.30	3.71	-16.41	3.86
	(299 to 392)	(71.01 to 156.53)	(3.24 to 4.25)	(-9.85 to 35.23)	(3.26 to 4.23)	(-31.01 to 2.70)	

Data in parentheses are 95% uncertainty intervals (95% UI).

In 2019, the age-standardized mortality rates from suicide were highest in the states of Rio Grande do Sul, Roraima, and Santa Catarina, while the lowest were reported in Rio de Janeiro, Pará, Alagoas, and the Federal District. Age-standardized mortality rates from suicide declined in Brazil overall and in most states in the South, Southeast, Midwest, and North regions between 1990 and 2019. Most of the states that showed an increase in the age-standardized mortality rates from suicide within this timeframe (Ceará, Piauí, Paraíba, Bahia) are located in the Northeast region (Table 1).

In 2019, 623,151 (95% UI: 590,135 to 679,259) YLLs were due to suicide in Brazil, a 35.03% (95% UI: 26.58 to 47.57) increase compared to 1990. The 2019 age-standardized YLL rate due to suicide was 265.25 (95% UI: 251.33 to 288.91) per 100,000 inhabitants, accounting for 1.59% (95% UI: 1.5 to 1.73%) of the overall YLLs in the country. Age-standardized and crude rates of YLLs due to suicide declined by -18.38% (95% UI: -23.23 to -10.87) and -7.24 (95% UI: -13.05 to 1.37) compared to 1990, respectively (Table 2).

**FIGURE 1:**
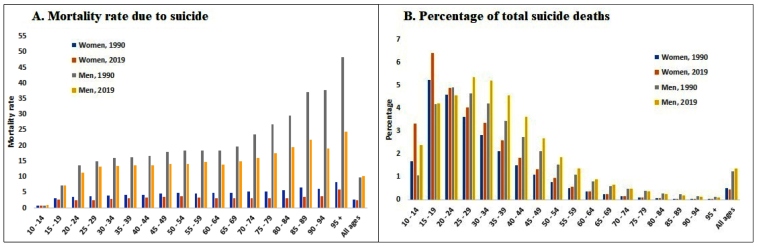
Mortality rate and percentage of total suicide deaths in males and females by age group in 1990 and 2019.

The 2019 age-standardized mortality rate for suicide was three to five times higher among men, regardless of the state (Table 1). Likewise, suicide mortality rates were higher among men, regardless of age, except for the 10 to 14 years of age group, in which rates were similar (Figure 1). 

**FIGURE 2:**
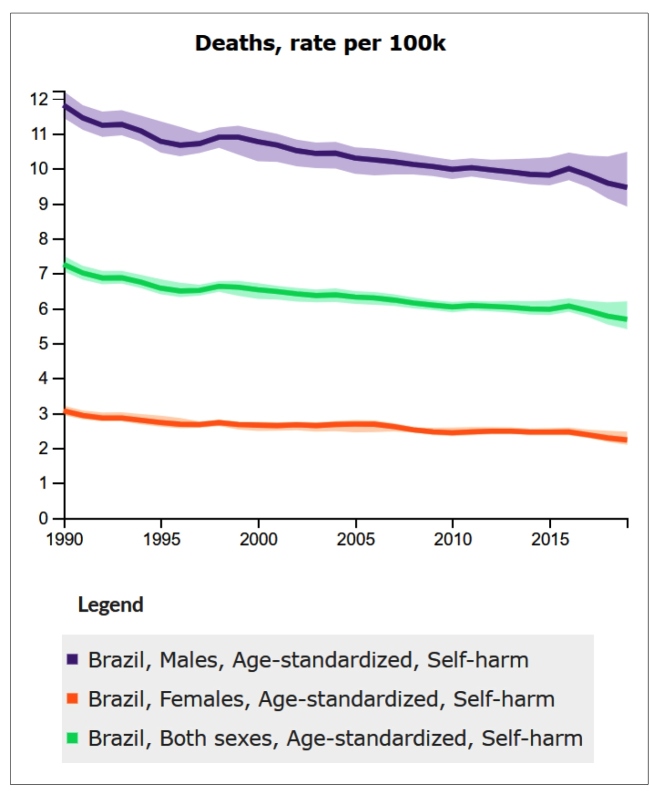
Annual estimates and uncertainty intervals of age-standardized mortality rate from suicide in Brazil, by sex, between 1990 and 2019.

**Source:** IHME, GBD Study Results Tool: http://ghdx.healthdata.org/gbd-results-tool

Figure 2 shows the annual estimates of age-standardized mortality rate due to suicide between 1990 and 2019, by sex. Suicide mortality rates were higher among men than women in all years. The age-standardized mortality rate due to suicide in women was 3.06 (95% UI: 2.94 to 3.21) and 2.23 (95% UI: 2.09 to 2.46) deaths per 100,000 population, in 1990 and 2019, respectively. For men, the age-standardized mortality rate due to suicide was 11.81 (95% UI: 11.45 to 12.21) in 1990 and 9.45 (95% UI: 8.91 to 10.47) deaths per 100,000 population, in 2019. The decline in suicide mortality rates from 1990 to 2019 was greater among women (-27.18%; 95% UI: -31.91 to -21.54) than among men (-19.95%; 95% UI: -25.28 to -11.23).

The mortality rates by age group showed an increasing gradient with aging, in 2019. From 1990 to 2019, suicide mortality rates declined among individuals over 15 years of age. In older individuals in particular, the decline in suicide mortality rates was four-fold greater than in younger individuals. Increase in suicide mortality rates (26.87%) were limited to the 10 to 14 years of age group, between 1990 and 2019. Still, 2019 suicide mortality rates were highest among individuals over 70 years of age (Table 2; **Figure 1**). 

**TABLE 2:** Total number of deaths, mortality rate, total number of years of life lost (YLL), and rate of YLL due to suicide in 2019, and total percent change from 1990 to 2019, by age group, for Brazil.

Age group (years)	Deaths				YLL			
	Number of deaths	% change	Mortality rates	% change	Number of YLL	% change	YLL rates	% change
	2019	1990-2019	2019	1990-2019	2019	1990-2019	2019	1990-2019
10-14	143	19.64	0.88	26.87	10 950	19.59	67.13	26.81
	(126 to 161)	(3.81 to 38.66)	(0.77 to 0.99)	(10.08 to 47.03)	(9 648 to 12 312)	(3.76 to 38.60)	(59.15 to 75.48)	(10.03 to 46.97)
15-19	816	3.53	4.96	-3.8	58 280	3.5	354.20	-3.83
	(745 to 894)	(-6.51 to 15.46)	(4.53 to 5.44)	(-13.13 to 7.28)	(53 217 to 63 831)	(-6.53 to 15.42)	(323.43 to 387.94)	(-13.15 to 7.25)
20-24	1 215	1.49	6.96	-18.7	80 917	1.55	463.23	-19.65
	(1 115 to 1 362)	(-8.65 to 14.44)	(6.39 to 7.80)	(-26.82 to -8.32)	(74 289 to 90 734)	(-8.6 to 14.52)	(425.29 to 519.44)	(-26.78 to 8.26)
25-29	1 334	13.32	7.78	-14.97	82 302	13.42	479.99	-14.9
	(1 233 to 1 458)	(1.01 to 26.60)	(7.19 to 8.51)	(-24.20 to -5.0)	(76 057 to 89 952)	(1.10 to 26.71)	(443.57 to 524.61)	(-24.14 to -4.92)
30-34	1 413	28.39	8.06	-18.52	80 071	28.34	456.80	-18.48
	(1 303 to 1 549)	(14.03 to 43.05)	(7.44 to 8.84)	(-27.63 to -9.21)	(73 850 to 87 781)	(14.07 to 43.12)	(421.31 to 500.79)	(-27.60 to -9.17)
35-39	1 445	52.49	8.25	-17.53	74 711	52.51	426.30	-17.52
	(1 329 to 1 591)	(36.73 to 71.13)	(7.59 to 9.08)	(-26.06 to -7.45)	(68 749 to 82 268)	(36.75 to 71.16)	(392.28 to 469.42)	(-26.05 to -7.44)
40-44	1 307	62.99	8.32	-19.54	61 128	62.99	389.27	-19.54
	(1 194 to 1 451)	(46.16 to 83.17)	(7.61 to 9.24)	(-27.84 to -9.58)	(55 867 to 67 853)	(46.16 to 83.17)	(355.76 to 432.09)	(-27.84 to -9.57)
45-49	1 175	70.85	8.60	-23.17	49 251	70.87	360.49	-23.16
	(1 080 to 1 313)	(54.51 to 93.71)	(7.91 to 9.62)	(-30.52 to -12.89)	(45 282 to 55 064)	(54.53 to 93.72)	(331.44 to 403.04)	(-30.51 to -12.88)
50-54	1 093	83.7	8.63	-25.18	40 633	83.62	320.51	-25.22	
	(1 012 to 1 213)	(66.25 to 125.42)	(7.99 to 9.57)	(-32.29 to -15.99)	(37 624 to 45 069)	(66.17 to 106.17)	(296.78 to 355.5)	(-32.32 to -16.03)	
55-59	972	102.56	8.67	-22.92	31 581	102.44	281.37	-22.97	
	(904 to 1 077)	(85.35 to 125.42)	(8.05 to 9.6)	(-29.47 to -14.22)	(29 347 to 34 984)	(85.24 to 125.30)	(261.46 to 311.69)	(-29.51 to -14.27)	
60-64	746	85.34	8.07	-28.34	20 800	85.22	224.93	-28.38	
	(691 to 819)	(69.87 to 105.19)	(7.47 to 8.86)	(-34.32 to -20.66)	(19 255 to 22 837)	(69.75 to 105.06)	(208.22 to 246.95)	(-34.36 to -20.71)	
65-69	612	92.34	8.51	-28.41	14 316	92.19	199.02	-28.47	
	(567 to 672)	(76.26 to 113.22)	(7.9 to 9.35)	(-34.40 to -20.64)	(13 279 to 15 717)	(76.12 to 113.06)	(184.61 to 218.5)	(-34.45 to -20.70)	
70-74	472	81.81	8.96	-34.76	9 029	81.51	171.20	-34.87	
	(434 to 522)	(65.69 to 105.06)	(8.24 to 9.91)	(-40.55 to -26.42)	(8 307 to 9 984)	(65.41 to 104.71)	(157.51 to 189.31)	(-40.65 to -26.55)	
75-79	330	72.9	9.27	-38.06	5 001	72.46	140.11	-38.21	
	(301 to 364)	(57.88 to 95.60)	(8.44 to 10.21)	(-43.44 to -29.92)	(4 553 to 5 505)	(57.45 to 95.12)	(127.58 to 154.24)	(-43.59 to -30.09)	
80-84	224	106.11	9.77	-38.45	2 601	105.24	113.25	-38.71	
	(195 to 247)	(87.42 to 133.80)	(8.5 to 10.76)	(-44.03 to -30.18)	(2 264 to 2 864)	(86.63 to 183.32)	(98.6 to 124.72)	(-44.27 to -30.47)	
85-89	127	142.64	10.40	-44.25	1 129	141.54	91.88	-44.5	
	(106 to 143)	(120.29 to 177.75)	(8.65 to 11.65)	(-49.39 to -36.18)	(939 to 1 265)	(119.27 to 176.49)	(76.44 to 102.98)	(-49.62 to 36.47)	
90-94	48	267.94	9.04	-49.01	328	265.57	61.79	-49.34	
	(36 to 54)	(228.95 to 317.48)	(6.97 to 10.32)	(-54.41 to -42.14)	(252 to 374)	(226.84 to 314.84)	(47.6 to 70.51)	(-54.70 to -42.51)	
95+	23	539.91	12.28	-42.49	115	502.57	61.63	-45.85	
	(16 to 27)	(467.22 to 614.45)	(8.9 to 14.4)	(-49.02 to -35.79)	(84 to 135)	(434.81 to 573.94)	(44.69 to 72.19)	(-51.94 to -39.43)	
All ages	13 503	46.00	6.23	0.32	623 151	35.03	28.76	-7.24	
	(12 815 to 14 735)	(37.20 to 59.87)	(5.91 to 6.80)	(-5.75 to 9.82)	(590 136 to 679 260)	(26.58 to 47.57)	(27.24 to 31.35)	(-13.05 to 1.37)	
Age- standardized			5.68	-21.68			265.25	-18.38	
			(5.4 to 6.19)	(-26.30 to -14.28)			(251.33 to 288.91)	(-23.23 to -10.87)	

Data in parentheses are 95% uncertainty intervals (95% UI).

**FIGURE 3:**
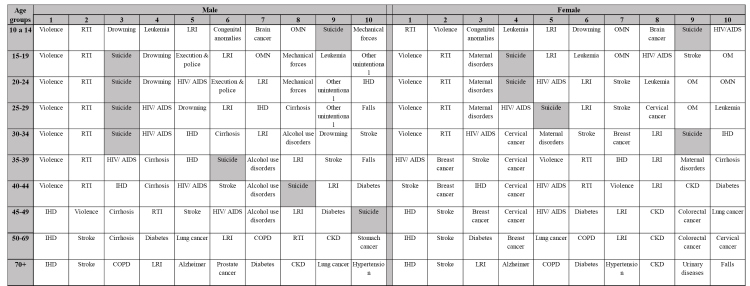
Leading ten causes of mortality in males and females by age group in Brazil, 2019.

**Note: CKD:** Chronic kidney disease; **COPD:** Chronic obstructive pulmonary disease; **IHD:** Ischemic Heart Disease; **LRI:** Lower Respiratory Infection; **OM:** Other musculoskeletal; **OMN:** Other malignant neoplasms; **RTI:** Road Traffic Injuries.

In 1990 and 2019, suicide numbers were highest (approximately two thirds of all deaths) among individuals of 15 to 49 years of age, regardless of sex, whereas suicide mortality rates were highest among older individuals over 70 years of age (Table 2; Figure 1). Nevertheless, in 2019, suicide was listed among the 10 leading causes of death in younger individuals but not in older adults of over 70 years of age. Suicide was one of the ten leading causes of death in men of 10 to 49 years of age, and women of 10 to 34 years of age in Brazil. It was also the third and fourth leading causes of death in men of 15 to 34 years of age and women of 15 to 24 years of age, respectively (Figure 3). 

## DISCUSSION

Rising numbers of suicide cases in Brazil over the last 30 years emphasize the need to a develop suicide prevention strategies. Suicide was among the 10 leading causes of death, for both sexes, in adolescents and young adults. Growing suicide rates among adolescents is a matter of concern and should be given priority in prevention policies. Suicide death rates differ widely according to sex and state. Age-standardized mortality rates due to suicide were higher among men, older adults, and South region residents. From 1990 to 2019, age-standardized mortality rates due to suicide declined in the South and increased in the Northeast. These changes may offset region-related disparities over time. Estimates provided in this study may support suicide prevention policies and contribute to suicide burden surveillance in different locations. 

Compared to global estimates, Brazil saw a less drop in age-standardized mortality rates for suicide from 1990 to 2019 (38.91% and 21.68%, respectively). Moreover, case numbers increased by 46% in the country compared to a 2.7% increase worldwide^8^. Therefore, family and social impacts of suicide were more dramatic in Brazil, where more than one third of the cases involved economically active individuals, leading to high individual and societal costs^11^. Growing suicide numbers suggest greater exposure to suicide risk factors, such as mental disorders, particularly depression, alcoholism, and schizophrenia, with direct impacts on health services^12^.

We also emphasize the reduction in age-standardized mortality rates due to suicide from 1990 to 2019, will probably not be sufficient to reach the SDG target of reducing suicide mortality rates by one third, from 2015 to 2030^4^. In recent years, Brazil has carried out some prevention actions, such as “September Yellow”, a campaign to make the population aware of the seriousness of the problem and help those in need. Additional studies are needed to assess the impact of suicide prevention campaigns and programs in Brazil^13^. Some studies from other countries have shown that the impact and effectiveness of campaigns on depression or suicide awareness have had modest results^14^. 

The epidemiology of suicide is multifactorial and includes social, psychological, and biological components^7^. Regional variations in suicide mortality in Brazil are consistent with findings of prior studies, reporting higher suicide rates in the South, Midwest, and Southeast, as compared to the North and Northeast regions of the country^15^ ^,^ ^16^.

Higher age-standardized mortality rates for suicide in Southern states may reflect a higher prevalence of depression^17^ or the presence of suicide-prone populations, such as farmers, older adults, and European immigrants^15^ ^,^ ^16^ in this region. In India, higher suicide rates in more developed regions of the country^18^ were also consistent with a higher prevalence of depression^19^. However, in Brazilian regions with lower socioeconomic status and limited access to services, such as the North and Northeast of the country, suicide mortality may be under-reported due to poorly equipped health facilities and low levels of public security, particularly in small to mid-sized municipalities^20^.

Recent age-standardized mortality rates for suicide estimates were expected to follow the overall downward trend in mortality in Brazil (37.92%)^8^. However, this study revealed a 21.69% decline in age-standardized mortality rates for suicide, with differences among states. 

In more developed regions of the country (Southern, Southeastern, and Midwest states), the decline in age-standardized mortality rates due to suicide may have reflected improved living conditions, as shown by the lower Gini index (a measure of income distribution), lower numbers of individuals with low income, or declining unemployment and illiteracy rates^21^. Likewise, the 64.1% decline in Chinese suicide rates between 1990 and 2016 was attributed to economic growth, urbanization, rising living standards, and higher access to medical care in rural areas^22^. 

The increase in the suicide rate in the last few years, particularly in Northeastern states, may have reflected socioeconomic changes and exposure of suicide risk factors, as violence^23^, and alcohol use^24^ or a refined definition of underlying causes of death listed in the SIM, particularly in the North and Northeast regions of the country^20^. In the latter case, increased rates may have resulted from enhanced case reporting. However, further studies with specific methodology are warranted to determine whether factors other than enhanced case reporting are associated with higher suicide rates in some states. 

This study revealed sizeable sex-related differences in suicide rates. Age-standardized mortality rates for suicide were higher among men in all states. This finding is congruent with data reported in previous national studies^7^ ^,^ ^15^ ^,^ ^16^ ^,^ ^25^ and reflects data from 194 of 195 countries investigated in the study, including Brazil^5^. Evidence suggests that suicidal attempts are more common among women but more successful among men, since men tend to use more lethal methods^19^ ^,^ ^26^. Women are also more likely to seek help to overcome mental disorders and suicidal ideations, are less prone to alcoholism, and tend to have stronger religious beliefs^15^. The decline in suicide rates was greater among Brazilian women when compared to Brazilian men (-23% and -13%, respectively) from 1990 to 2019. Similar global trends have also been reported^5^. Therefore, gender-related disparities may be expected to increase in the future.

Similar to other countries worldwide^5^, suicide rates were highest among older adults of over 70 years of age in Brazil. However, rising numbers of suicide cases among young people have had a greater impact on YLLs. These higher proportions of suicide deaths among those aged under 40 years reflects the age structure of the Brazilian population, since age-specific mortality rates in the under 40-year individuals are relatively lower compared with rates at older ages. 

As in the present study, Jean-Varas et al. (2019)^25^ reported a 24% increase in suicide rates in adolescents of 10 to 19 years of age, in six large Brazilian cities, and a 13% increase in Brazil overall, between 2006 and 2015. Likewise, a study conducted by Fernandes et al. (2020)^27^ showed that mortality rates, from 1997 to 2016, increased by 1.35% per year among adolescents. These findings emphasize the need for expanding care services for children and adolescents, which are still insufficient in Brazil^28^ and planning specific prevention interventions for this stage of life^29^ ^,^ ^30^.

A systematic review and meta-analysis of studies investigating the impact of interventions that were specifically designed to reduce suicide-related behavior in young people concluded these interventions can reduce self-harm and suicidal ideation. Prevention programs were developed in clinical, school and community settings. The authors suggested further research adapted to online suicide prevention interventions to reach this audience. This study also highlighted few investigations like this one have been conducted in low- and middle-income countries, such as Brazil^30^.

Although WHO recognizes suicide as a public health priority, most countries, including Brazil, understand suicide as a minor event. This might be explained by the fact suicide rates in Brazil are considered low as compared to other countries. In addition, mortality from suicide is lower compared to other types of deaths from external causes, leading policy makers to direct most of their resources to combating these deaths^23^. In Brazil, only a small proportion of its health budget is allocated to mental health policies^31^. 

In Brazil, government actions started only in 2006, when the National Guidelines for Prevention of Suicide were launched^32^. Other important initiatives included the enactment of a law, in 2019, which established the National Policy for Prevention of Self-Mutilation and Suicide, involving the participation of civil society and private health and education organizations in promoting mental health for prevention of self-mutilation and suicide^33^. These are important measures to improve official suicide statistics, given that adequate recording and regular monitoring of suicide at the national level are the bases of effective prevention strategies^3^. 

Given causes of suicide in Brazil are often associated with socioeconomic factors, such as unemployment and social inequality^21^ ^,^ ^25^, macroeconomic policies are warranted to reduce suicide rates in the population. Promote protection factors, wider availability, and higher access to mental health services^7^ and awareness campaigns aimed to mitigate stigmatization and prevent alcohol and drug abuse may also play an important role in suicide prevention^3^. Specific low-cost strategies that have proven effective in other countries include reducing access to the means of suicide, through revised legislation on gun ownership, development of media guidelines on responsible reporting of these deaths, online self-help to reduce suicidal ideation, and community involvement that can regularly keep contact with people who have attempted suicide, and support persons bereaved by suicide^2^. 

Methodological limitations of the GBD study regarding suicide death estimations are primarily related to under-reporting and misclassification of suicide, which occur due to social, cultural, religious, and even legal factors^5^. To overcome this bias, some death codes were reclassified in the GBD study in order to provide more accurate estimates. This is a strength of the data presented in this study, as it improves the estimates. However, deaths by suicide may also be reported as death by other plausible causes, such as unintentional injuries, which were not accounted for in the GBD reclassification process. In this case, GBD estimates may be viewed as conservative. 

Another limitation of this study is the lack of information about other potential risk factors for suicide in the country, such as marital status, socioeconomic status, education, mental illness, ethnicity, violence, alcohol use, among others. The lack of these data limits the development of prevention strategies suited to the country's regional characteristics. In addition, suicide methods that would be useful in designing specific preventive interventions were not presented.

In conclusion, age-standardized mortality rates and YLL rates from suicide have declined in Brazil in recent decades. However, suicide remains a significant public health concern. A continued focus on strengthening data on suicide deaths is required, as well as data on means of suicide. Suicide qualifies a preventable cause of death. Findings of this study can be used by public policy makers to formulate suicide prevention strategies. Suicide prevention should be encouraged, as well as efforts focused on more vulnerable groups and regions, such as males, older adults, and young people living in the South region of the country.

Analysis of suicide data from all states may help design prevention plans tailored to each state. Adopted interventions must be continuously monitored, and the outcomes communicated to enable the replication of successful strategies to different contexts.
